# Effect of Different Cleaning Methods on Shear Bond Strength of Resin Cement to Contaminated Zirconia

**DOI:** 10.3390/ma15145068

**Published:** 2022-07-21

**Authors:** Maher Saeed Hajjaj, Saeed Jamaan Alzahrani

**Affiliations:** Department of Restorative Dentistry, Faculty of Dentistry, King Abdulaziz University, Jeddah 21589, Saudi Arabia; sjalzahrani1@kau.edu.sa

**Keywords:** contamination, zirconia cleaning, zirconia bonding, bond strength

## Abstract

The aim of this study was to evaluate the effect of different cleaning methods on the shear bond strength (SBS) of resin cement to contaminated zirconia specimens. Eighty rectangular-shaped specimens (2 × 5 × 10 mm) were fabricated from Zirconia blocks (IPS e.max ZirCAD) and randomly divided into 8 groups (*n* = 10). Group A (control) was not exposed to contaminants. The following tests specimens were contaminated with saliva and silicone indicating paste. Group B was coated with ceramic primer, then subjected to contamination. Groups C, D, E, F, G, and H were contaminated; cleaned with water rinse, Ivoclean, air particle abrasion, hydrofluoric acid, KATANA^TM^ Cleaner and ZirClean^TM^, respectively, and then coated with ceramic primer and bonded to dual cure resin cement cylinders. All the specimens were subjected to artificial aging and surviving specimens were subjected to the SBS test. For statistical analysis, ANOVA and multiple comparison methods at the 0.05 significance level were used. There was no statistically significant difference among Ivoclean (21.48 ± 2.90 MPa), air particle abrasion (21.92 ± 2.85 MPa), and the control group (24.68 ± 5.46). The application of ceramic primer before contamination did not preserve the SBS of resin cement to zirconia. Cleaning the contaminated zirconia surface with hydrofluoric acid (15.03 ± 3.63) or KATANA^TM^ Cleaner (17.27 ± 7.63) did not restore SBS to the uncontaminated state, but it was significantly higher than simply rinsing with water (12.46 ± 5.17) or the use of ZirClean^TM^ (11.59 ± 5.53). The bond strength of resin cement to zirconia was influenced by cleaning methods.

## 1. Introduction

There has been a significant increase in the use of zirconia-based restorations due to their high mechanical properties, excellent biocompatibility, ease of fabrication, and acceptable esthetics [1,2]. The longevity of the zirconia restoration is influenced by the durability of the bond strength between the luting cement to the zirconia surface, as well as to the tooth structure. Unlike glass ceramic material, etching zirconia to create micromechanical retention is not achievable with hydrofluoric acid due to the lack of glass phase [3,4,5]. Bonding zirconia to the tooth surface with resin cement involves two interfaces. The strong adhesive interface is between enamel/dentin and resin cement, and the weaker adhesive interface is between zirconia and resin cement [5,6].

In addition, exposure to contaminants (blood, saliva, or silicone indicating paste) during the clinical try-in of indirect restorations is inevitable. Contamination negatively affects the quality of the bond strength between the resin cement and the zirconia surface [7,8,9]. Rinsing with water is insufficient to decontaminate the bonding surface due to the adherence of organic material of saliva after contamination [10,11]. Thus, adequate cleaning of the contaminated zirconia surface is necessary for the longevity of the treatment, particularly for restorations with deficient retention and resistance forms.

Cleaning the contaminated surface with simple water rinse showed ineffective results in restoring the bond strength [12]. Several methods for cleaning contaminated zirconia before bonding have been reported in the literature [13,14,15,16,17,18,19,20]. Mechanical cleaning via sandblasting with alumina particles (Al_2_O_3_) is used to clean contaminated surfaces and increase surface energy, which effectively restores the original bond strength [13,14,15]. When sandblasting zirconia, it is recommended to follow adequate parameters for particle size, distance, and pressure to avoid large surface flaws [16].

Chemical cleaning methods for contaminated zirconia have also been reported. They are safely applied without an adverse effect on the mechanical properties of zirconia. Phosphoric acid has been effective in removing organic contaminants, but X-ray photoelectron spectroscopy proves that the presence of phosphorous residue which might influence the bonding negatively [17]. Hydrofluoric acid is readily available in the clinic and has been used to clean contaminated zirconia surfaces; it is believed that no residue of hydrofluoric acid remains when using this method in comparison to phosphoric acid [18]. Several commercially available products are advocated, for example, chemical cleaners for dental zirconia, such as Ivoclean (Ivoclar Vivadent, Schaan, Liechtenstein), ZirClean^TM^ (BISCO, Inc., Schaumburg, IL, USA), or [11] KATANA^TM^ Cleaner (Kurary Noritake Dental Inc., Okayama, Japan). Ivoclean is a highly alkaline (pH = 13) solution applied extraorally on zirconia surfaces for cleaning before cementation. This is a ceramic cleaner that has been shown to effectively clean contaminated surfaces [19,20]. This material contains zirconium oxide; when it is applied to a contaminated zirconia surface, it attracts contaminants, which is followed by water rinsing and air-drying to leave a clean surface [13,21]. ZirClean^TM^ is also an alkaline cleaning gel composed of potassium hydroxide, which breaks the ionic bond formed between the zirconia surface and the organic material from saliva [13]. KATANA^TM^ Cleaner is a mild acidic (pH = 4.5) cleaner containing 10-methacryloyloxdecal dihydrogen phosphate (10-MDP) salt and can be used for intraoral or extraoral applications. 10-MDP has a hydrophobic group that bonds to contaminants and a hydrophilic group that makes it easily removed by water rinse [13]. Another promising technique is Non-Thermal Atmospheric Plasma (NTAP) [22]. Despite this method of cleaning being difficult to be applied clinically, it has shown effective results in elimination of saliva [23].

Before bonding, it is recommended to apply a chemical promotor as a ceramic primer that contains 10-MDP on the zirconia surface to enhance the bonding to resin cements [24]. 10-MDP is an organophosphate monomer with two functional groups—a phosphate group that reacts with zirconia and a methacrylate group that copolymerizes with the resin cement [14]. Hence, the idea of 10-MDP ceramic primer application before exposure to contaminants was suggested as a preventive measure to preserve bond strength [20]. Thermocycling is a simulated ageing procedure that is used to stress the adhesive bond by exposing the specimens to different water tanks with different temperatures [17].

Recently introduced cleaning materials, such as KATANA^TM^ Cleaner and ZirClean^TM^, have been tested in dry conditions [13,25], and it is recommended to investigate these materials after artificial ageing with thermocycling [13,26]. Considering the difficulty of zirconia etching and the negative influence of contaminants on the zirconia surface after clinical try-in steps, this in vitro study aimed to evaluate the shear bond strength of resin cement to contaminated zirconia specimens after using recently introduced surface cleaning solutions (Ivoclean, KATANA^TM^ Cleaner and ZirClean^TM^) with conventional techniques (water rinse, air particle abrasion, or hydrofluoric acid). In addition, to test the impact of applying 10-MDP ceramic primer prior to contamination, as recommended by Angkasith et al. [20] The first null hypothesis is that there is no significant difference in the shear bond strength of resin cement and contaminated zirconia after using different cleaning methods. The second null hypothesis is that the application of ceramic primer prior to contamination will preserve the bond strength of resin cement to zirconia.

## 2. Materials and Methods

Pre-sintered zirconia blocks (IPS e.max ZirCAD, Ivoclar Vivadent, Schaan, Liechtenstein) were sectioned into eighty 2 × 5 × 10 mm specimens using a low-speed diamond saw (Allied techcut 4Low Speed Diamond Saw, Rancho Dominguez, CA USA) under water coolant. All specimens were polished with multiple silicon carbide papers (320, 400, 600, and 1200 grits) at 300 rpm for 20 s each. Then, specimens were sintered in a Vita ZYrcomat T furnace (Vita Zahnfabrik, Bad Sackingen, Germany) according to the manufacturer’s instructions. Specimens were embedded in chemically polymerized acrylic resin (poliREPAR S, Polident) for handling purposes during the shear bond strength test. The bonding surfaces of the specimens were inspected for any acrylic flashes and polished using 1200 grit carbide paper to remove all residues. Specimens were subjected to air particle abrasion with 50 µm Aluminum oxide (Al_2_O_3_) powder at 2.0 bar and at a 10 mm distance for 20 s. (Duostar Sandblaster, Bego, Bremen, Germany). Later, they were ultrasonically cleaned in distilled water for 5 min, then dried with a stream of air.

**Figure 1:**
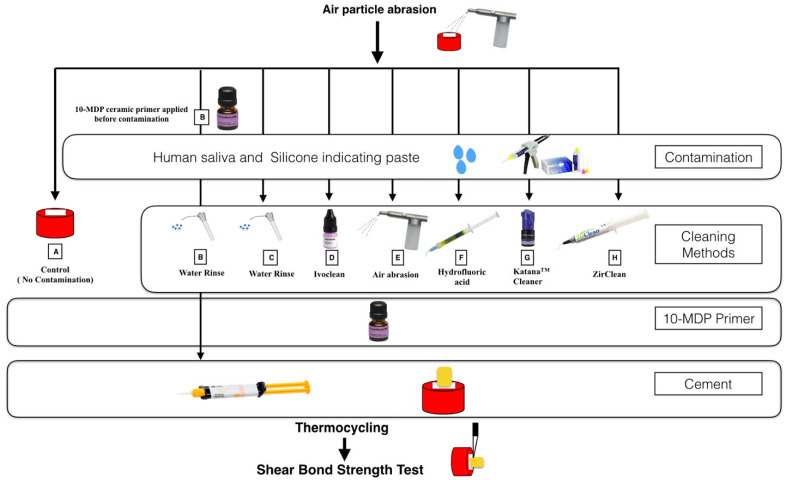
Flowchart of the test groups and cleaning methods.

**Figure 2:**
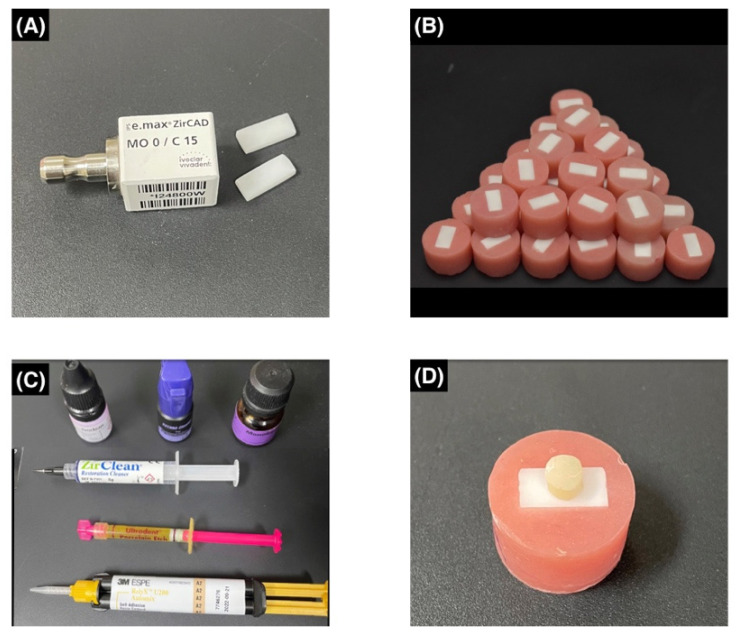
Steps of specimens’ preparation: (**A**) Cutting of rectangular-shaped pre-sintered zirconia blocks (IPS e.max ZirCAD, Ivoclar Vivadent, Schaan, Liechtenstein); (**B**) Embedded zirconia in acrylic resin; (**C**) Materials used for surface cleaning and bonding; and (**D**) Resin cement cylinder bonded to zirconia surface.

After that, specimens were randomly divided into 8 groups (*n* = 10) according to the cleaning methods. In the control group (group A), 10-MDP ceramic primer (Monobond N, Ivoclar Vivadent, Schaan, Liechtenstein) was applied using a micro-brush with agitation for 10 s and left for 60 s and then excess primer was dispersed using a dry-air stream for 5 s. For contamination, saliva was collected from a healthy male individual who refrained from eating, drinking, and brushing for 2 h before the collection process (Ethical approval from Research Ethics Committee, Faculty of Dentistry, King Abdulaziz University #175-12-20). In group B, specimens were coated with 10-MDP ceramic primer before exposure to contaminants, followed by contamination with human saliva, which was applied to zirconia surface and spread with a micro-brush for 2 min before rinsing with water and drying. Then, inorganic contamination was carried out by applying a layer of silicone indicating paste (Fit Checker Advanced Blue cartridge pack, GC America, Alsip, IL, USA) under 1 kg load and it was removed from the surface once it set (3 min). In groups C, D, E, F, G, and H, zirconia specimens were contaminated with saliva and silicone indicating paste, as mentioned above. Cleaning protocols were performed as described in the following. In Group C, specimens were only rinsed with water for 15 s and dried. In Group D, Ivoclean was applied using a micro-brush and left to react for 20 s and then rinsed with water thoroughly and dried. In Group E, specimens were subjected to additional air particle abrasion with 50 µm Aluminum oxide (Al_2_O_3_) powder at 2.0 bar and at a 10 mm distance for 20 s, then rinsed with water and dried. In Group F, specimens were treated with 9.6% hydrofluoric acid for 30 s and then rinsed with water and dried. In Group G, KATANA^TM^ Cleaner was applied to the specimens and rubbed using a micro-brush for 10 s, then rinsed with water and dried. In group H, a thin layer of ZirClean^TM^ was applied using a delivery tip, left for 20 s, and then rinsed with water and dried. A split Teflon mold was placed on the zirconia surface to produce a cylinder with the following dimensions: 4.25 mm diameter and 4 mm height. All specimens were dried with a dry-air stream then coated with 10-MDP ceramic primer as described earlier (Monobond N, Ivoclar Vivadent, Schaan, Liechtenstein). Self-adhesive resin cement (RelyX^TM^ U200 automix, 3M ESPE, Seefeld, Germany) was injected into the mold and light cured for 40 s using an E-Morlit curing light (Apoza, NewTaipei, Taiwan) delivering a power of 1200 mW/cm^2^. The mold was disassembled carefully after light curing and any cement flashes were carefully removed using a sharp scalpel. Then, specimens were subjected to 5000 thermal cycles of artificial ageing at a temperature between 5 and 55 °C, which represents a 6-month intraoral simulation (Thermocycler THE-1100, Mechatronik, Pleidelsheim, Germany). Each cycle took about 1 min to complete. Table 1 summarizes the types and compositions of materials used in this study, Figure 1 demonstrates the flowchart of the test groups and cleaning methods and Figure 2 demonstrates steps of specimen preparation.

**Figure 3:**
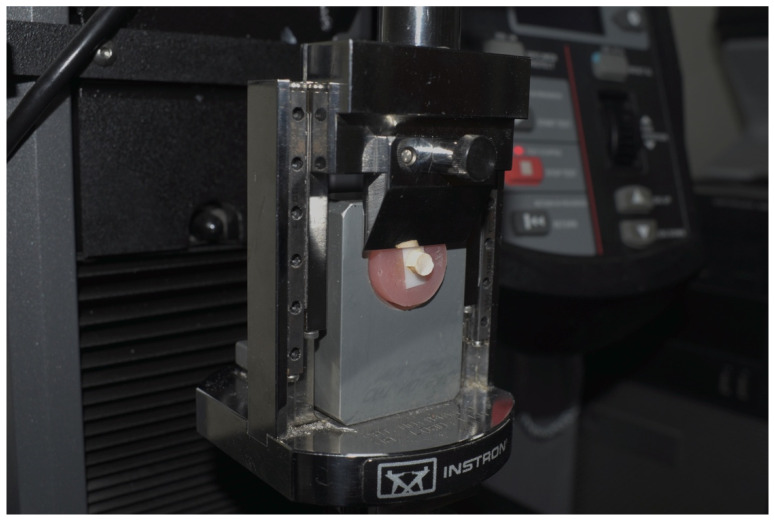
Specimen mounted in universal testing machine for shear bond strength test.

Shear bond strength (SBS) was measured using the universal testing machine (INSTRON, Norwood, MA, USA). The load was applied to the interface of the zirconia-resin cement at a crosshead speed of 1 mm/min until failure (Figure 3).

All the data were subjected to statistical analysis. Statistical analysis was performed using Statistical Package for the Social Sciences SPSS (version 20), while Microsoft Office Excel 2010 was used for data handling and graphical presentation. One-way analysis of variance (ANOVA) was used to compare the mean shear bond strength of different groups. The post hoc tests used Bonferroni method for comparison between tests groups. To compare each group with the control group, Dunnett’s test was used. The result was considered significant at a *p*-value of < 0.05. Two-tailed tests were assumed throughout the analysis for all statistical tests.

## 3. Results

Out of 80 specimens, 64 survived thermocycling. Three groups did not have any specimens that failed after thermocycling: control (A), Ivoclean (D), and air abrasion (E) groups. On the other hand, all the B group specimens, which were treated by applying primer only before contamination, failed during thermocycling.

**Figure 4:**
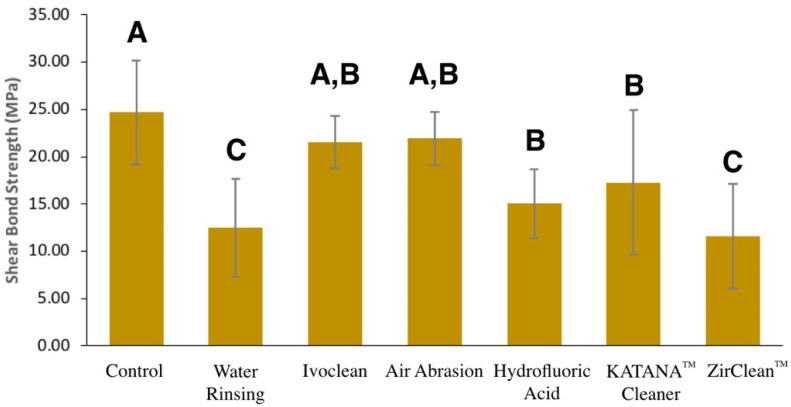
Shear bond strength of self-adhesive resin cement to saliva and silicone indicating paste-contaminated zirconia that had been exposed to different cleaning methods. Data are mean ± standard deviations. Vertical bars labeled with the same letter indicate no significant difference (*p* > 0.05).

The mean shear bond strength values in MPa and standard deviation were as follows: the control group A (24.68 ± 5.46), water rinsing group C (12.46 ± 5.17), Ivoclean group D (21.53 ± 2.74), air particle abrasion group E (21.92 ± 2.85), hydrofluoric acid group F (15.03 ± 3.63), KATANA^TM^ Cleaner group G (17.27 ± 7.63), and ZirClean^TM^ group H (11.59 ± 5.53). Table 2 and Figure 4 summarize the descriptive statistics for all groups.

The Shapiro–Wilk test was used to test the normality hypothesis of all the quantitative variables for a further choice of appropriate parametric and non-parametric tests. All the variables were found to be normally distributed, allowing the use of parametric tests. Levene’s test of homogeneity of variance was also applied. A one-way ANOVA showed highly statistically significant differences between the groups (*p* < 0.001), as presented in Table 3.

The first null hypothesis that there is no significant difference in the shear bond strength of resin cement to contaminated zirconia after using different cleaning methods was rejected. The application of 10-MDP ceramic primer before contamination (group B) did not preserve the SBS and all the specimens failed during thermocycling, so the second null hypothesis was also rejected.

Multiple comparisons between test groups were performed with the Bonferroni post hoc test. The result showed that the mean SBS of Ivoclean and air particle abrasion groups were significantly higher than water rinsing and ZirClean^TM^ groups, but they were statistically similar to hydrofluoric acid and KATANA^TM^ Cleaner groups. Zirclean^TM^ group was the weakest among all groups and it was statistically similar to water rinsing. To compare each test group with the control group, Dunnett’s test showed that the SBS of control group was statistically similar to that of the Ivoclean and air particle abrasion groups, but significantly higher than that of the rest of the groups (water rinsing, hydrofluoric acid, KATANA^TM^ Cleaner, and ZirClean^TM^ groups).

## 4. Discussion

The durability of the bonding of zirconia restorations is important for long-term success [27]. In the present in vitro study, we evaluated the shear bond strength of resin cement to saliva and silicone indicating paste contaminated zirconia specimens after decontamination with water rinsing, Ivoclean, air particle abrasion, hydrofluoric acid, KATANA^TM^ Cleaner, and ZirClean^TM^. Exposure to contaminants significantly influence the bond strength (*p* < 0.05). Only Ivoclean and air particle abrasion were able to restore the shear bond strength of resin cement to zirconia to the uncontaminated (control) state. The first null hypothesis that there is no significant difference in the shear bond strength of resin cement to contaminated zirconia after using different cleaning methods was rejected (*p* < 0.001). We also tested the hypothesis that the application of 10-MDP ceramic primer prior to contamination will preserve the bond strength, this hypothesis was also rejected, as none of the specimens survived after artificial ageing.

The application of ceramic primer containing 10-MDP on the cleaned zirconia surface is recommended for increasing bond strength [7]. The phosphate ester group of the 10-MDP reacts with the zirconia surface to create a bond that is more resistant to biodegradation and increases the durability of the bonding [22,26]. The mechanism of chemical interaction of 10-MDP and zirconia were investigated by Nagaoka et al. [28]. In addition to an ionic bond, they found that there is a hydrogen bond that is formed between 10-MDP and zirconia.

Angkasith et al. [20] recommended the application of 10-MDP ceramic primer to the try-in step to maintain the bond strength without using any cleaning agent. In the current study, this seemed to be an ineffective method, because none of the specimens survived after 5000 thermal cycles. This indicates that water rinsing after contamination with saliva and silicone indicating paste was not enough to clean the contaminated zirconia surface. However, applying 10-MDP ceramic primer after contamination alone did restore half of the bond strength (12.46 MPa) of the control group (24.67 MPa). The highest shear bond strength in the test groups was found in the air particle abrasion (21.92 MPa) and Ivoclean (21.53 MPa) cleaning method group. Mechanical cleaning with air particle abrasion has proven to be a successful method for cleaning contaminated surfaces [7,9,13,17]. This method increases the surface area and surface energy to enhance the bonding to the zirconia surface. The influence of the application of air particle abrasion on mechanical properties to create a flaw in the surface has been investigated previously, with contradictory results [29,30,31,32]. Ivoclean is a chemical cleaning method and has been reported as an effective way to restore shear bond strength after contamination [9,13,19,20,21]. It has affinity to organic contaminants higher than the zirconia surface due to the high concentration of the zirconia particles in the solution. KATANA^TM^ Cleaner is an acidic material (pH = 4.5) containing 10-MDP salts that act as a surfactant to remove hydrophobic contaminants [10]. It was introduced to the market as a cleaner for either intraoral or extraoral application and either for tooth or restorative materials [33]. ZirClean^TM^ is a ceramic cleaner solution containing potassium hydroxide with high alkalinity (pH > 13), which neutralizes acids in organic contaminants and cleans them [11]. In the current study, KATANA^TM^ Cleaner was more effective in restoring the SBS (17.27 MPa), while ZirClean^TM^ showed the lowest performance amongst all groups (11.59 MPa). There are limited studies available in the literature about the effectiveness of KATANA^TM^ Cleaner or ZirClean^TM^ as cleaning methods [13,22,25,26]. A single study included both ZirClean^TM^ and KATANA^TM^ Cleaner and found no significant difference in SBS [13]. However, we found a significant difference between KATANA^TM^ Cleaner and ZirClean^TM^. In our study, we used thermocycling to perform artificial ageing for 5000 cycles, which was not used in the aforementioned study, which might explains the different results [13]. Sahin et al. [26] found that after 1 week of ageing at 37 °C, there was no statistical difference in SBS between the Ivoclean and KATANA^TM^ Cleaner groups with two different resin cements. Awad et al. [22] and Tian et al. [11] found that Ivoclean and KATANA^TM^ cleaners are effective methods for the decontamination of a zirconia surface exposed to saliva and blood. In our study, we found a statistically significant difference between Ivoclean and KATANA^TM^ cleaners after thermocycling. Among the reasons for this difference is the use of different ceramic primers and resin cements in all the studies. In a meta-analysis [6], it was found that the type of cement used significantly influences the bond strength. Hydrofluoric acid is routinely used to etch glass ceramics to increase bond strength. However, due to the polycrystalline nature of zirconia, it is difficult to etch zirconia surfaces with 9.6% hydrofluoric acid. Hydrofluoric acid was applied to contaminated zirconia to dissolve organic contaminants from the surface, but the result was not satisfying (15.03 MPa) [13]. The simplest and most direct way of cleaning in the clinical setting is rinsing with water before applying 10-MDP. In the presented study, this method was proven to be ineffective in restoring bond strength. The same findings were reported by different authors, regardless of the contaminants used [11,13,22].

One of the limitations of our study was using a shear bond test instead of a microtensile bond test, which has more uniform stress distribution at the bonding interface. However, the shear bond test is commonly used for the assessment of ceramic bond strength and provides an acceptable ranking of the test groups. Another limitation is that specimens were not analyzed under a microscope to study the original, contaminated, and treated zirconia surfaces, or to categorize the failure mode.

## 5. Conclusions

From this in vitro study, the following conclusions were drawn. The contamination of zirconia restoration with saliva and silicone indicating paste significantly reduced the shear bond strength of resin cement to zirconia. Ivoclean and air particle abrasion were able to restore the SBS of resin cement to zirconia, similar to the uncontaminated control group. The application of ceramic primer to the zirconia surface before contamination did not preserve the SBS of resin cement to zirconia. Cleaning the contaminated zirconia surface with KATANA^TM^ Cleaner or hydrofluoric acid did not restore SBS to the uncontaminated state, but it was significantly higher than simply rinsing with water or the use of ZirClean^TM^. Artificial ageing with thermocycling is important to stress the bond strength and validate the bond strength between zirconia and resin cement.

## Figures and Tables

**Table 1:** Summary of the products used in the study.

Materials	Manufacturer	Composition
IPS e.max ZirCAD	Ivoclar-Vivadent AG, Schaan, Liechtenstein	ZrO_2_, Y_2_O_3_, HfO_2_, Al_2_O_3_, and other oxides
Monobond N	Ivoclar-Vivadent AG, Schaan, Liechtenstein	Alcohol solution of silane methacrylate, phosphoric acid methacrylate, sulfide methacrylate
RelyX U200 Automix	3M ESPE, Dental Products, Saint Paul, MN, USA	Base: Methacrylate monomers containing phosphoric acid groups, methacrylate monomers, silanated fillers, initiator components, stabilizer, rheological additives Catalyst: methacrylate monomers, alkaline (basic) fillers, silanated fillers, initiator components, stabilizer, pigments, rheological additives
Ivoclean	Ivoclar-Vivadent AG, Schaan, Liechtenstein	Polyethyleneglycol, sodium hydroxide, ZrO_2_, water
KATANA^TM^ Cleaner	Kuraray Noritake Dental Inc., Tokyo, Japan	Triethanolamine, polyethyleneglycol, 10-Methacryloyloxydecyl dihydrogen phosphate salt
ZirClean^TM^	Bisco Inc., Schaumburg, IL, USA	Potassium hydroxide
Korox 50	BEGO, Bremen, Germany	Al_2_O_3_ (50 μm)

**Table 2:** Descriptive statistics for shear bond strength test.

Group	Cleaning Method	N	Mean (MPa)	95% Confidence Interval for Mean	
				Lower Bound	Upper Bound
Group A (Control)	no contamination, primer	10	24.68 ± 5.46 ^A^	20.77	28.58
Group B	primer, contamination, water	0	All specimens failed during thermocycling		
Group C	contamination, water, primer	8	12.46 ± 5.17 ^C^	8.14	16.78
Group D	contamination, Ivoclean, primer	10	21.53 ± 2.74 ^A,B^	19.57	23.49
Group E	contamination, air abrasion, primer	10	21.92 ± 2.85 ^A,B^	19.89	23.96
Group F	contamination, hydrofluoric acid, primer	8	15.03 ± 3.63 ^B,C^	12.00	18.06
Group G	contamination, KATANA^TM^ Cleaner, primer	9	17.27 ± 7.63 ^B,C^	11.40	23.14
Group H	contamination, ZirClean^TM^, primer	9	11.59 ± 5.53 ^C^	7.34	15.84

Similar superscript letters indicate no statistically significant difference (*p* < 0.05).

**Table 3:** One-way ANOVA table.

	SS	df	MS	F	*p* Value
Between Groups	1413.801	6	235.634	9.602	<0.001 *
Within Groups	1398.796	57	24.540		
Total	2812.598	63			

ANOVA, analysis of variance; df, degree of freedom (*n*−1); MS, mean squares; SS, sum of squares. * Significant at *p* < 0.05.
